# Supplementary figures and images for: A stable microtubule bundle formed through an orchestrated multistep process controls quiescence exit
Source: eLife. 2024 Mar 25;12:RP89958. doi: 10.7554/eLife.89958 (PMC10963028; doi:10.7554/eLife.89958)

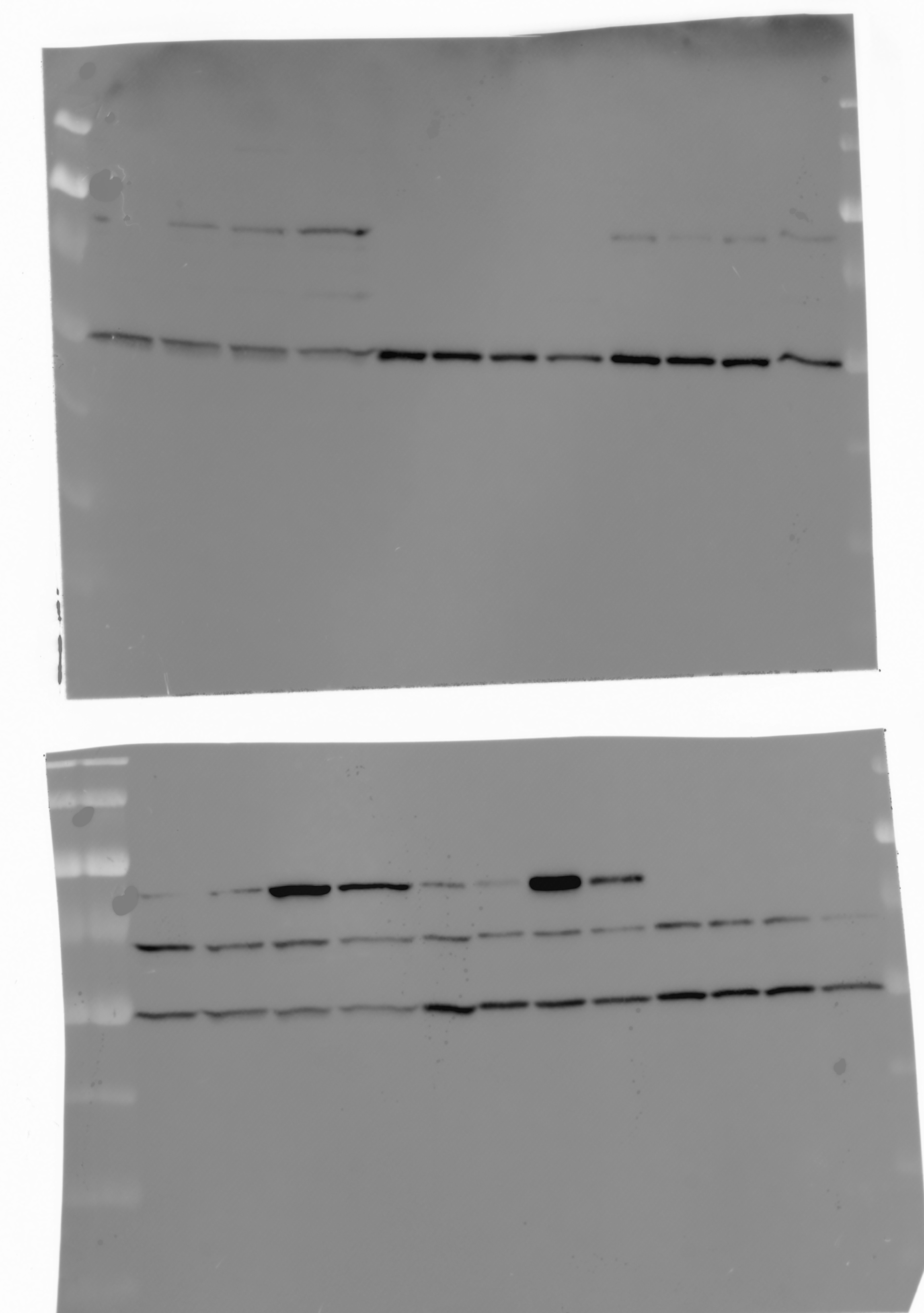

Supplement: Figure 1—figure supplement 1—source data 5. [file elife-89958-fig1-figsupp1-data5.zip › Figure1 Supplement 1H ade13 dual Western blot.tif]

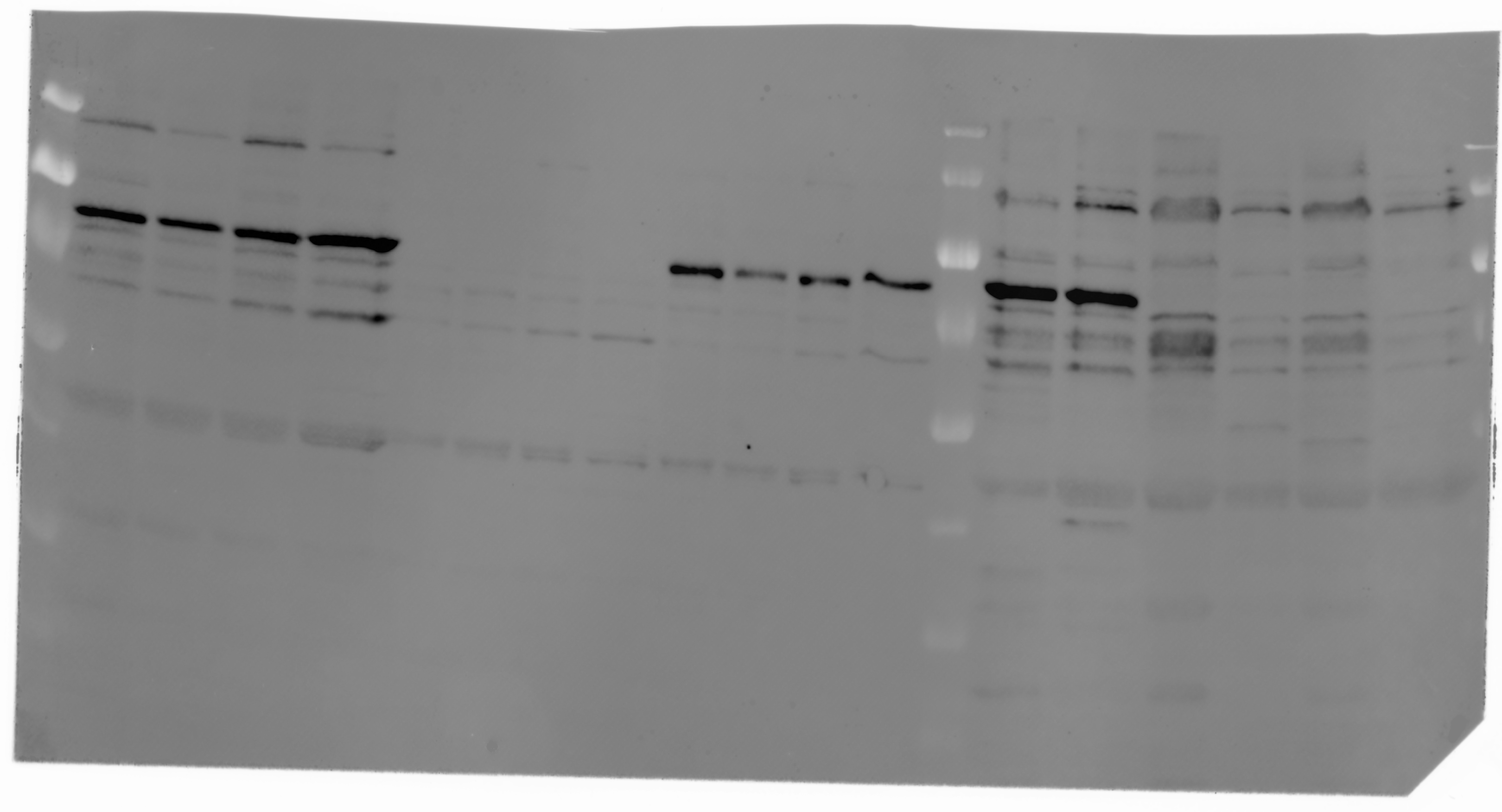

Supplement: Figure 1—figure supplement 1—source data 6. [file elife-89958-fig1-figsupp1-data6.zip › Figure1 Supplement 1H GFP dual Western blot.tif]

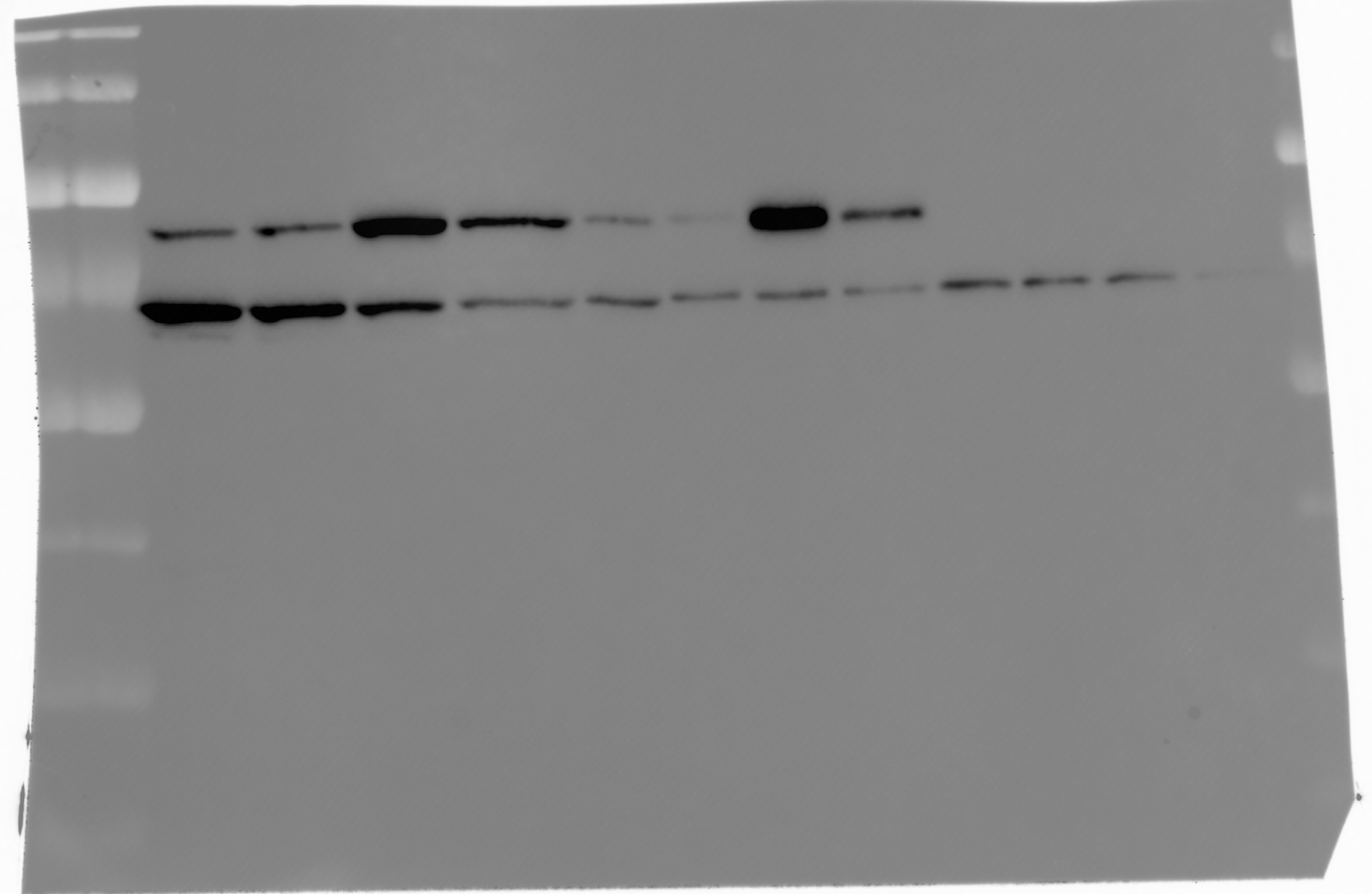

Supplement: Figure 1—figure supplement 1—source data 7. [file elife-89958-fig1-figsupp1-data7.zip › Figure1 Supplement 1H rfp dual Western blot.tif]

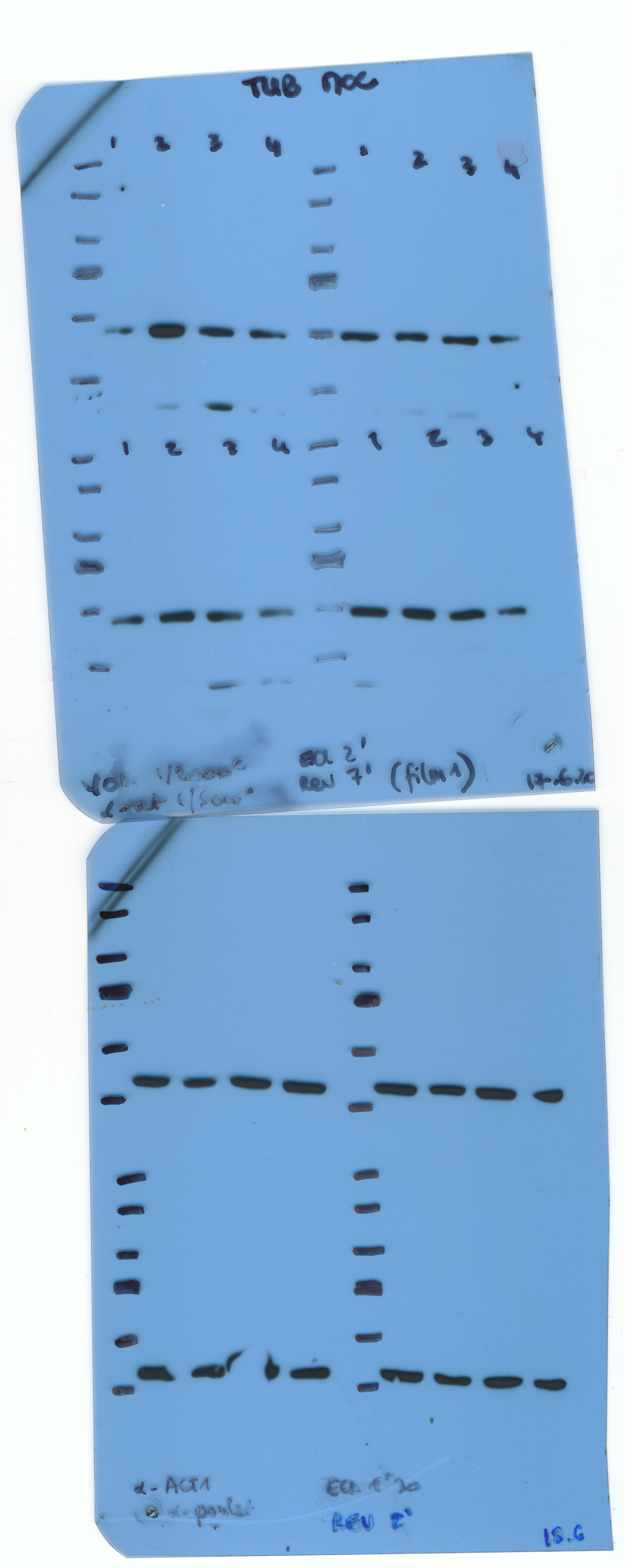

Supplement: Figure 2—figure supplement 1—source data 4. [file elife-89958-fig2-figsupp1-data4.zip › Figure 2 Supplemental 1B Western blot.tif]

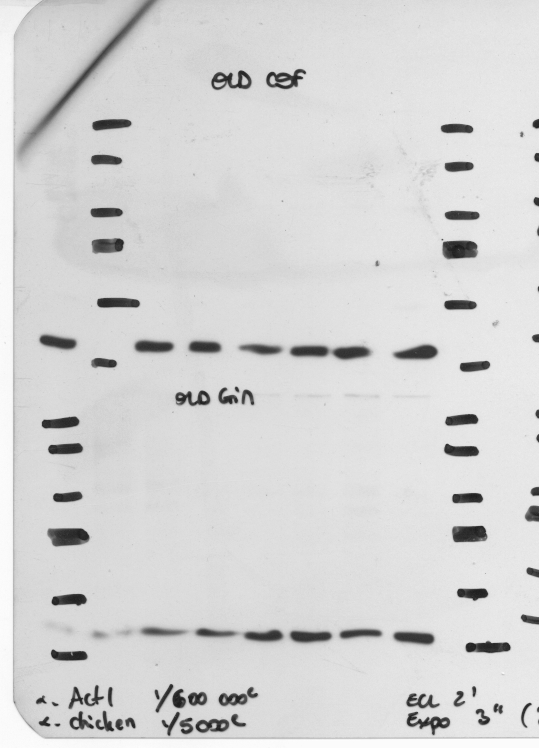

Supplement: Figure 2—figure supplement 1—source data 5. [file elife-89958-fig2-figsupp1-data5.zip › Figure 2 Supplemental 1D Western blot1.tif]

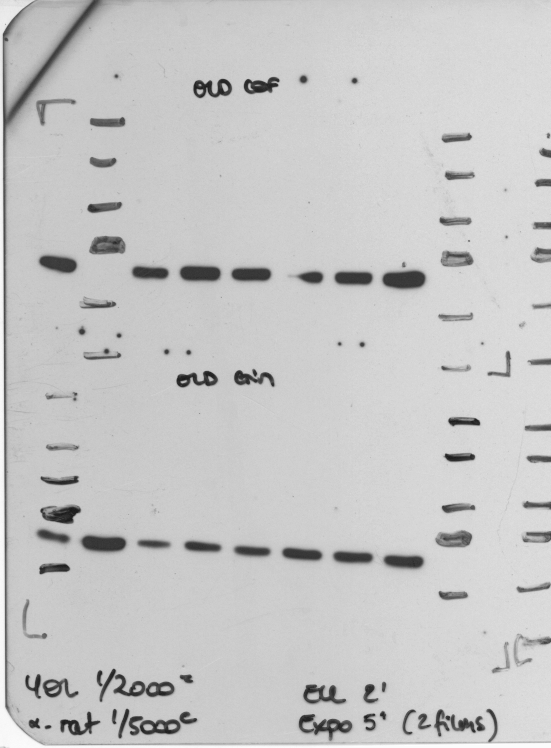

Supplement: Figure 2—figure supplement 1—source data 6. [file elife-89958-fig2-figsupp1-data6.zip › Figure 2 Supplemental 1D tub Western blot1.tif]

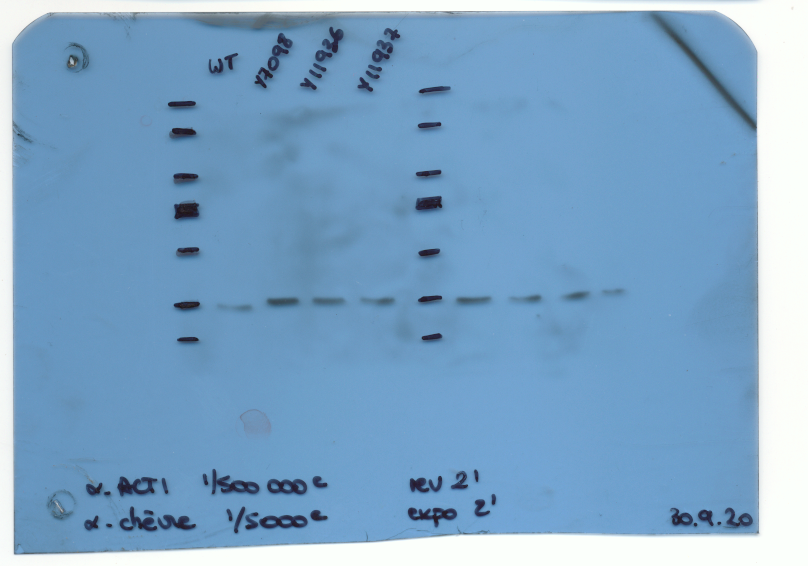

Supplement: Figure 2—figure supplement 1—source data 7. [file elife-89958-fig2-figsupp1-data7.zip › Figure 2 Supplemental 1E Western blot.tif]

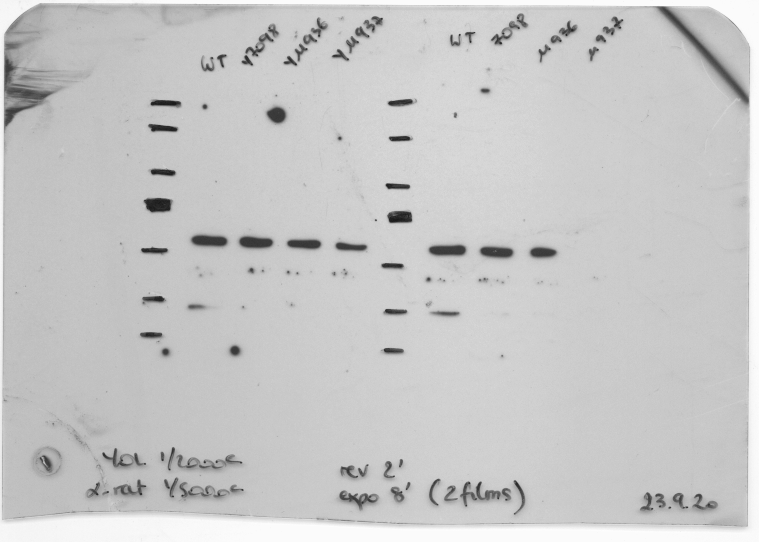

Supplement: Figure 2—figure supplement 1—source data 8. [file elife-89958-fig2-figsupp1-data8.zip › Figure 2 Supplemental 1E tub Western blot.tif]

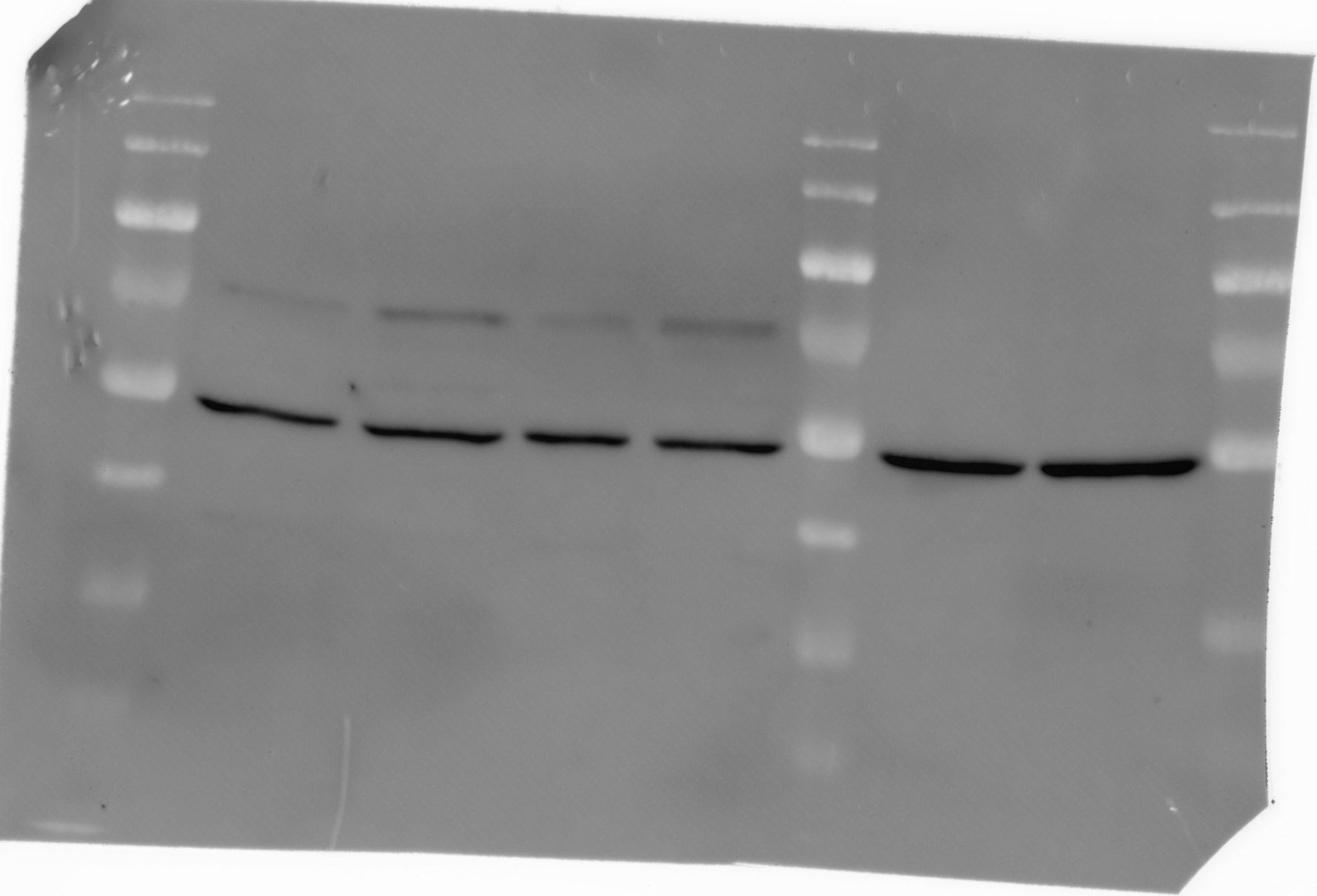

Supplement: Figure 3—figure supplement 1—source data 6. [file elife-89958-fig3-figsupp1-data6.zip › Figure3 Supplement 1E Ade13 Western blot.tif]

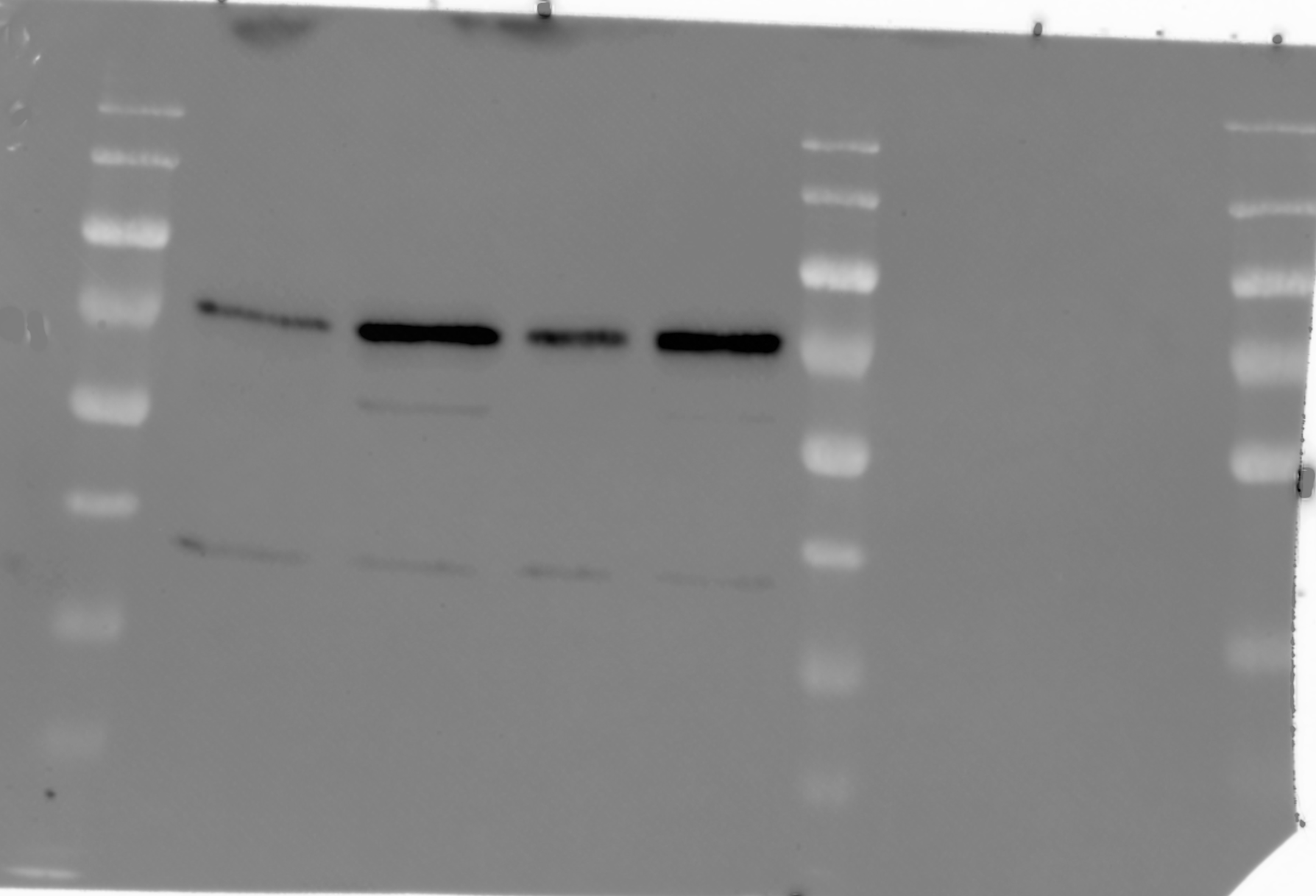

Supplement: Figure 3—figure supplement 1—source data 7. [file elife-89958-fig3-figsupp1-data7.zip › Figure3 Supplement 1E myc Western blot.tif]

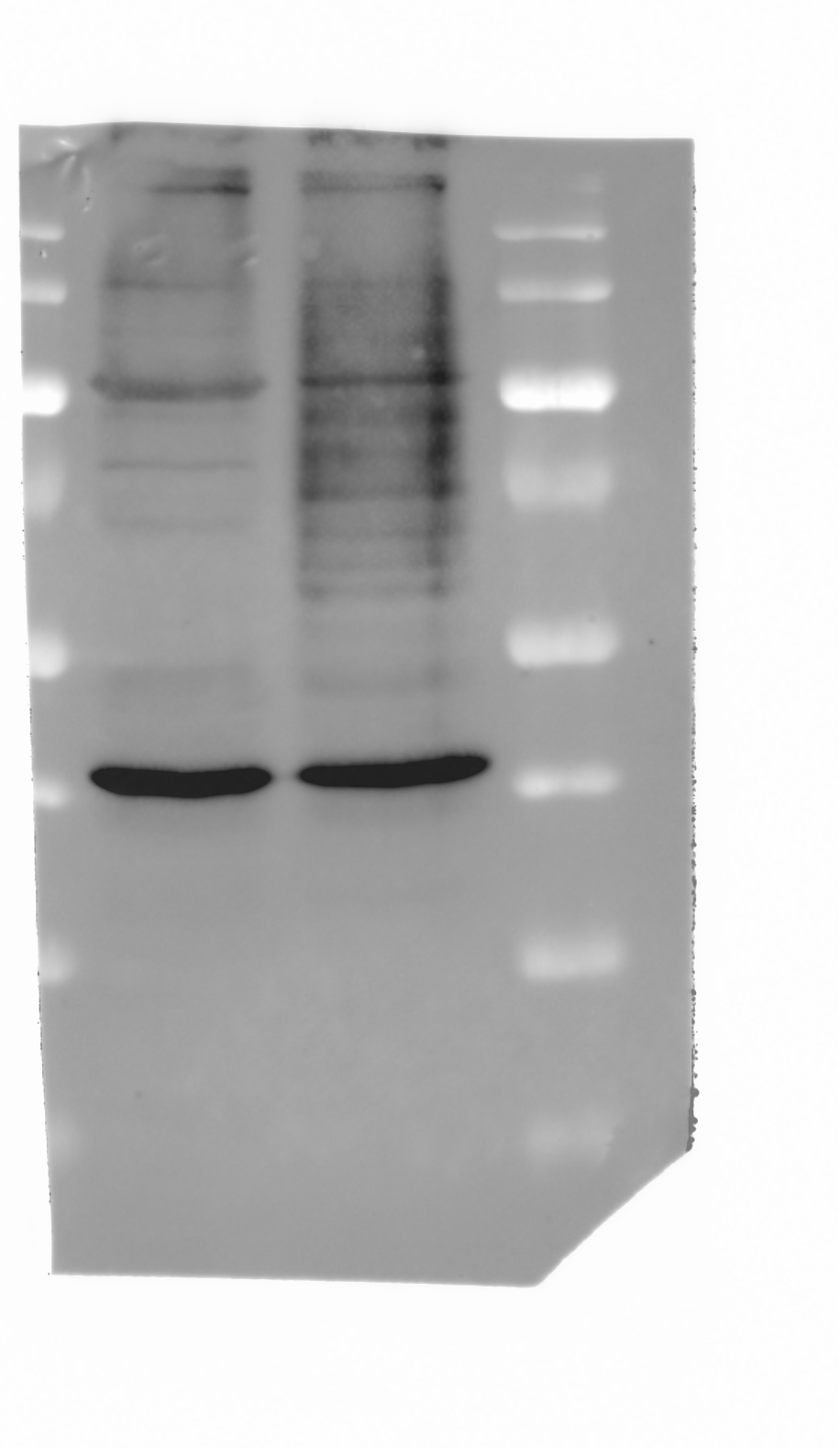

Supplement: Figure 3—figure supplement 1—source data 8. [file elife-89958-fig3-figsupp1-data8.zip › Figure3 Supplemental 1I Csm1act1 Western blot.tif]

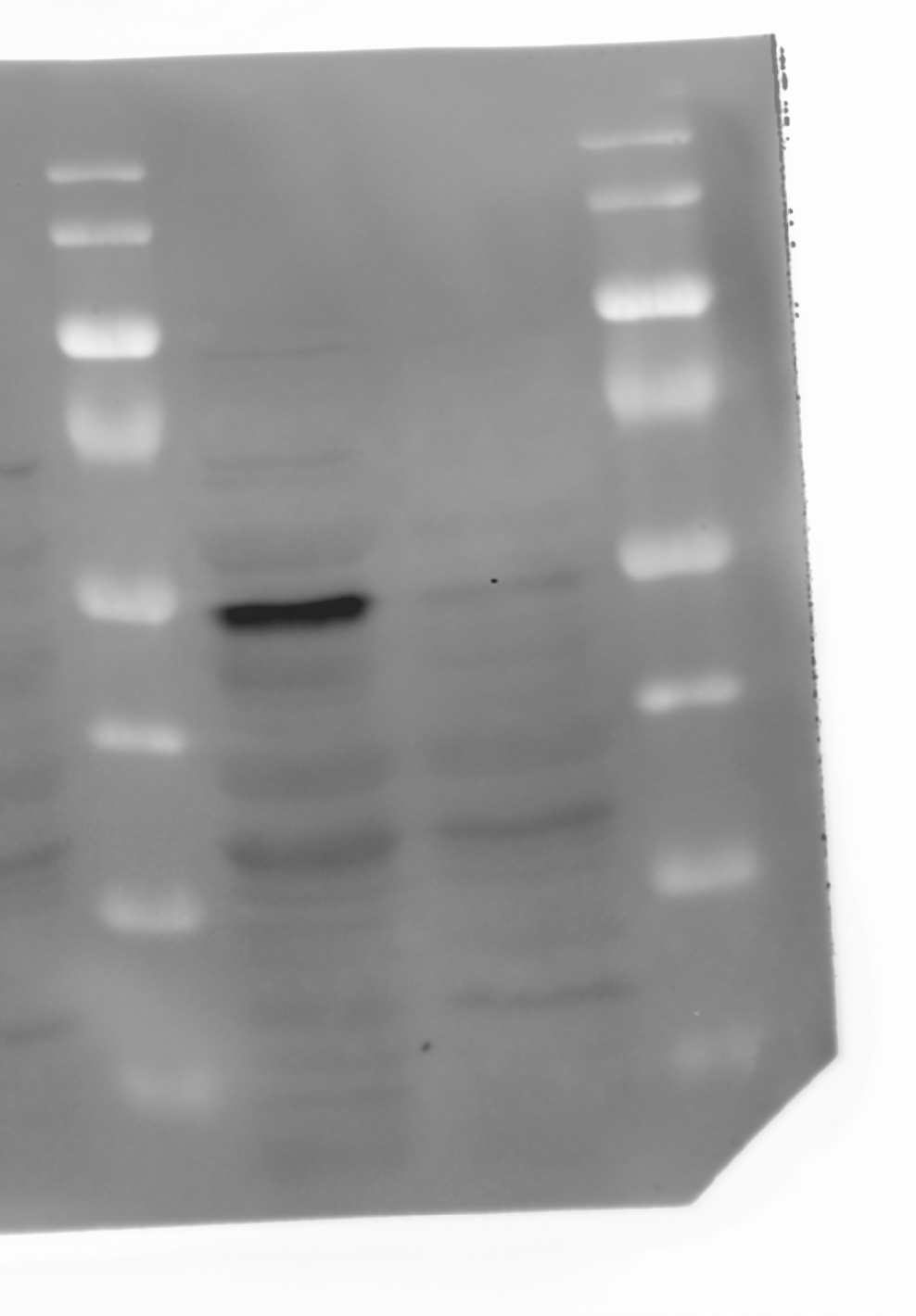

Supplement: Figure 3—figure supplement 1—source data 9. [file elife-89958-fig3-figsupp1-data9.zip › Figure3 Supplemental 1I Csm1GFP Western blot.tif]

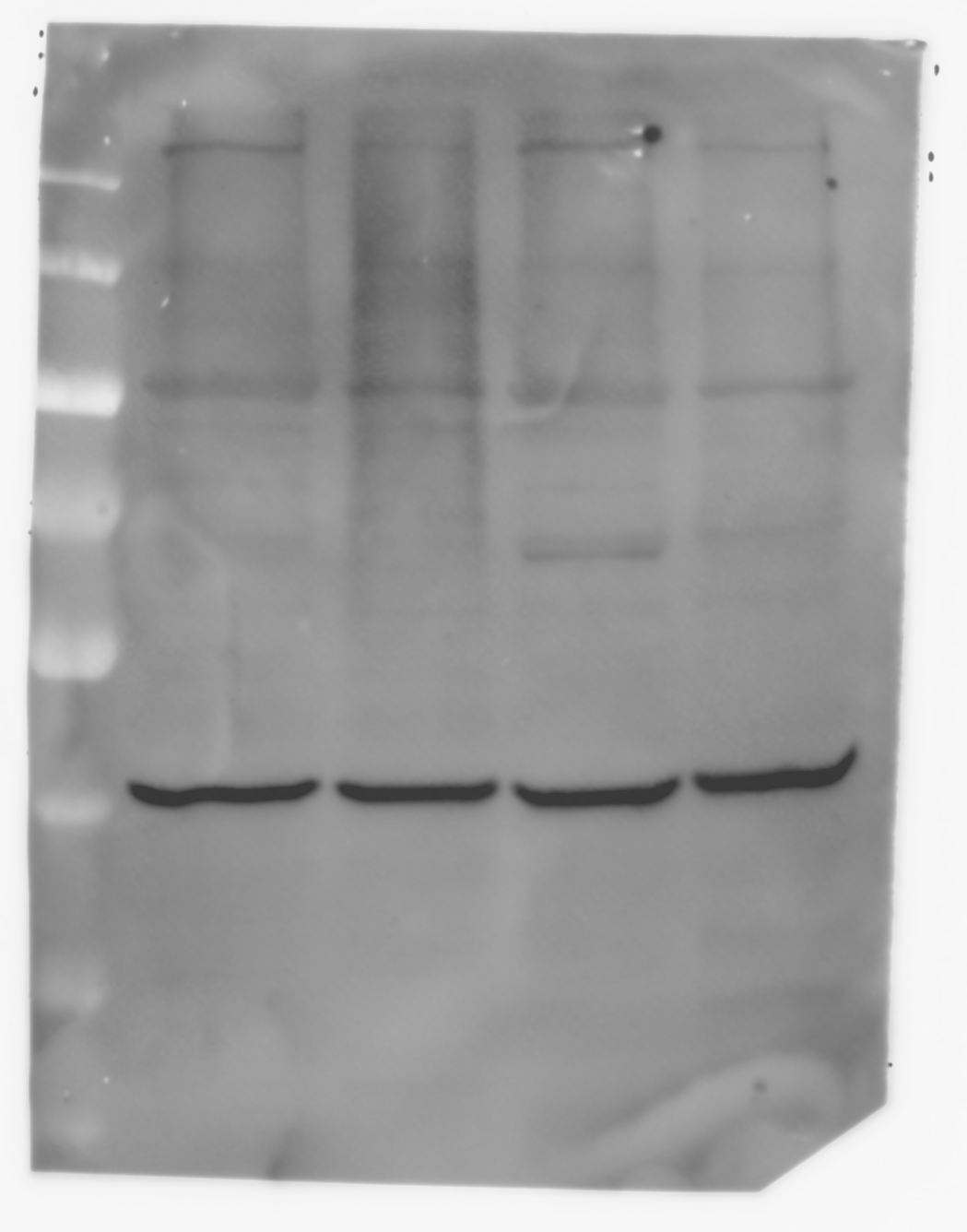

Supplement: Figure 3—figure supplement 1—source data 10. [file elife-89958-fig3-figsupp1-data10.zip › Figure3 Supplemental 1I Lrs4act1 Western blot.tif]

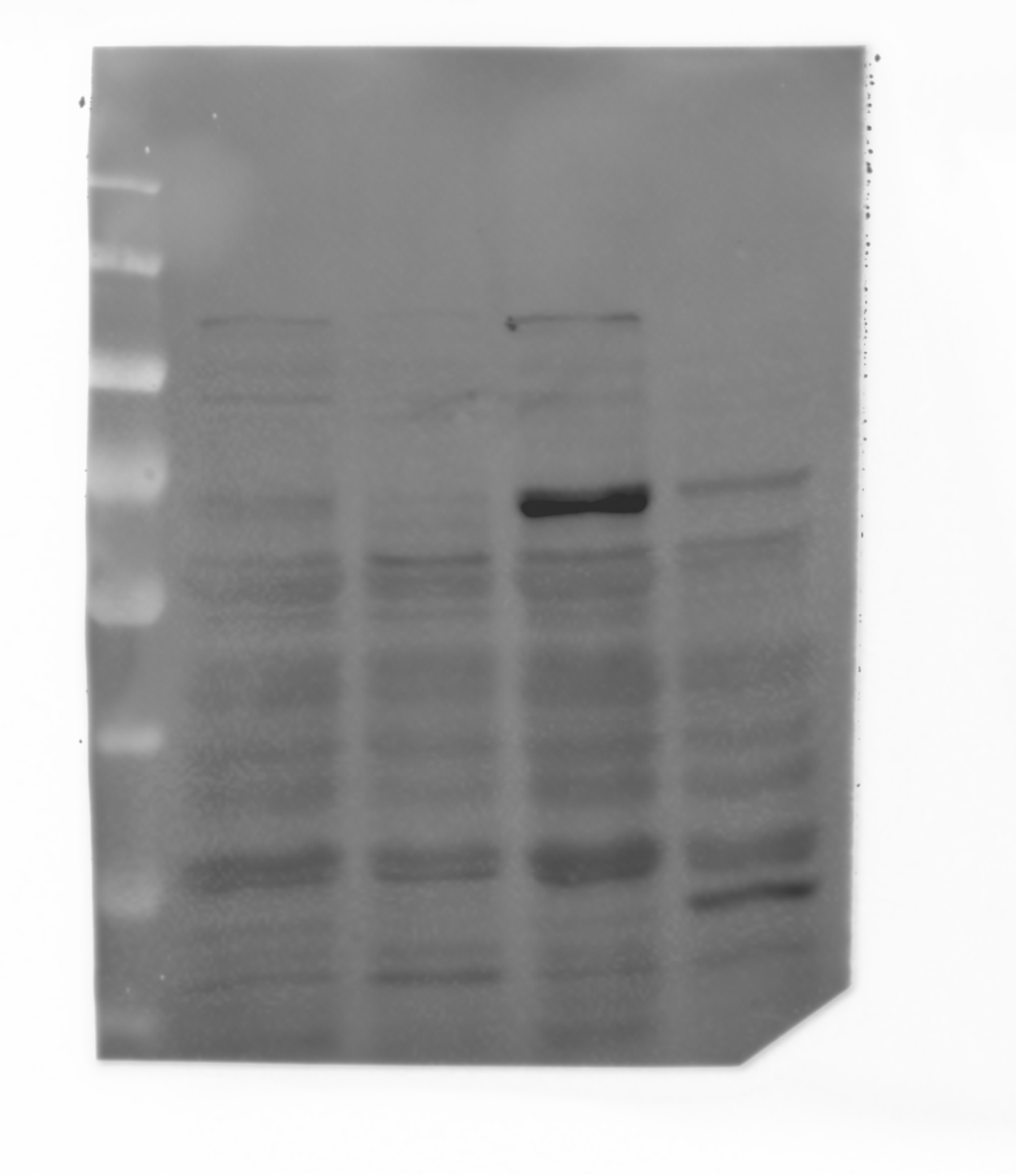

Supplement: Figure 3—figure supplement 1—source data 11. [file elife-89958-fig3-figsupp1-data11.zip › Figure3 Supplemental 1I Lrs4GFP Western blot.tif]

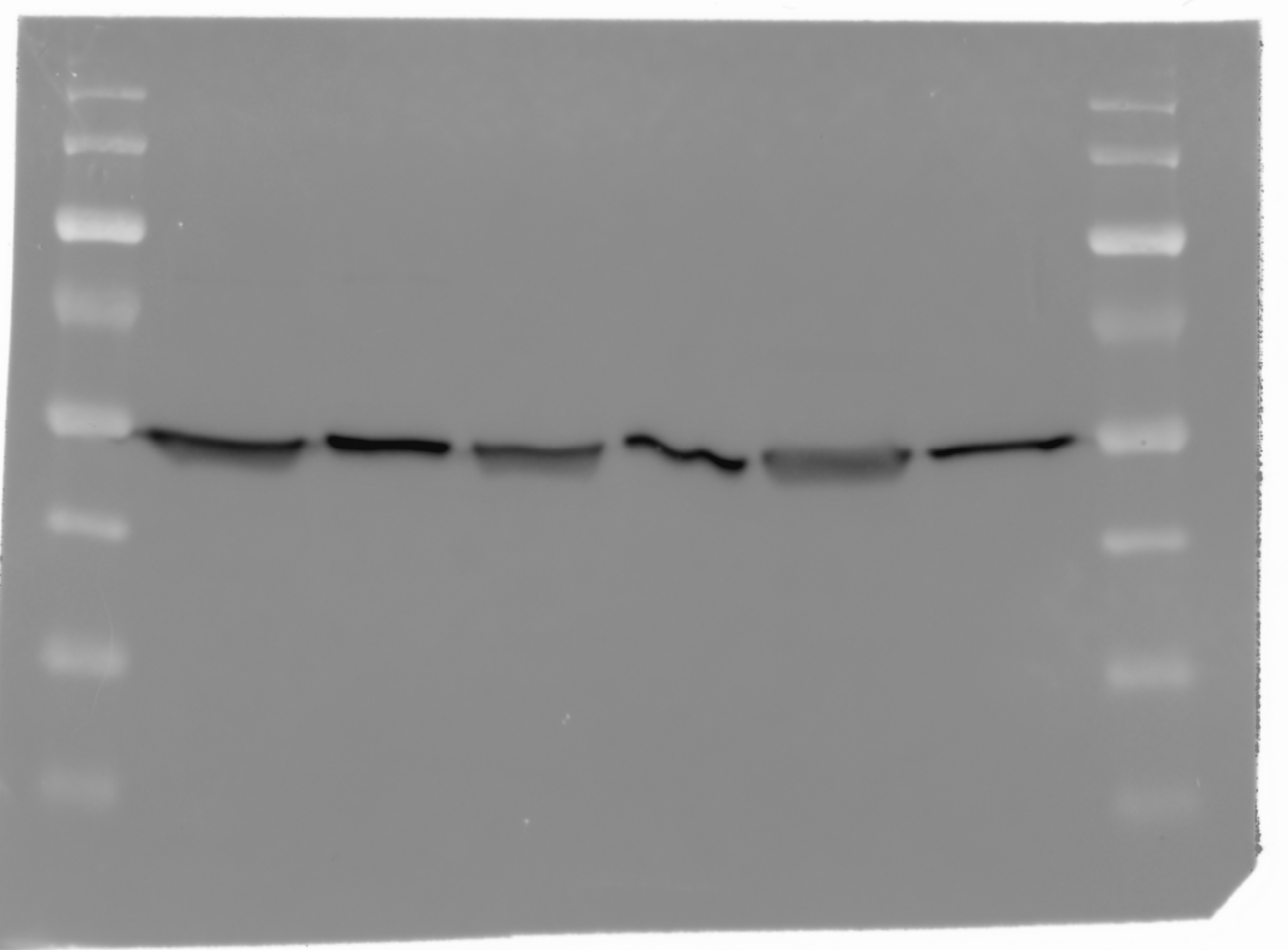

Supplement: Figure 3—figure supplement 1—source data 12. [file elife-89958-fig3-figsupp1-data12.zip › Figure3 Supplemental 1I Mam1ade13 Western blot.tif]

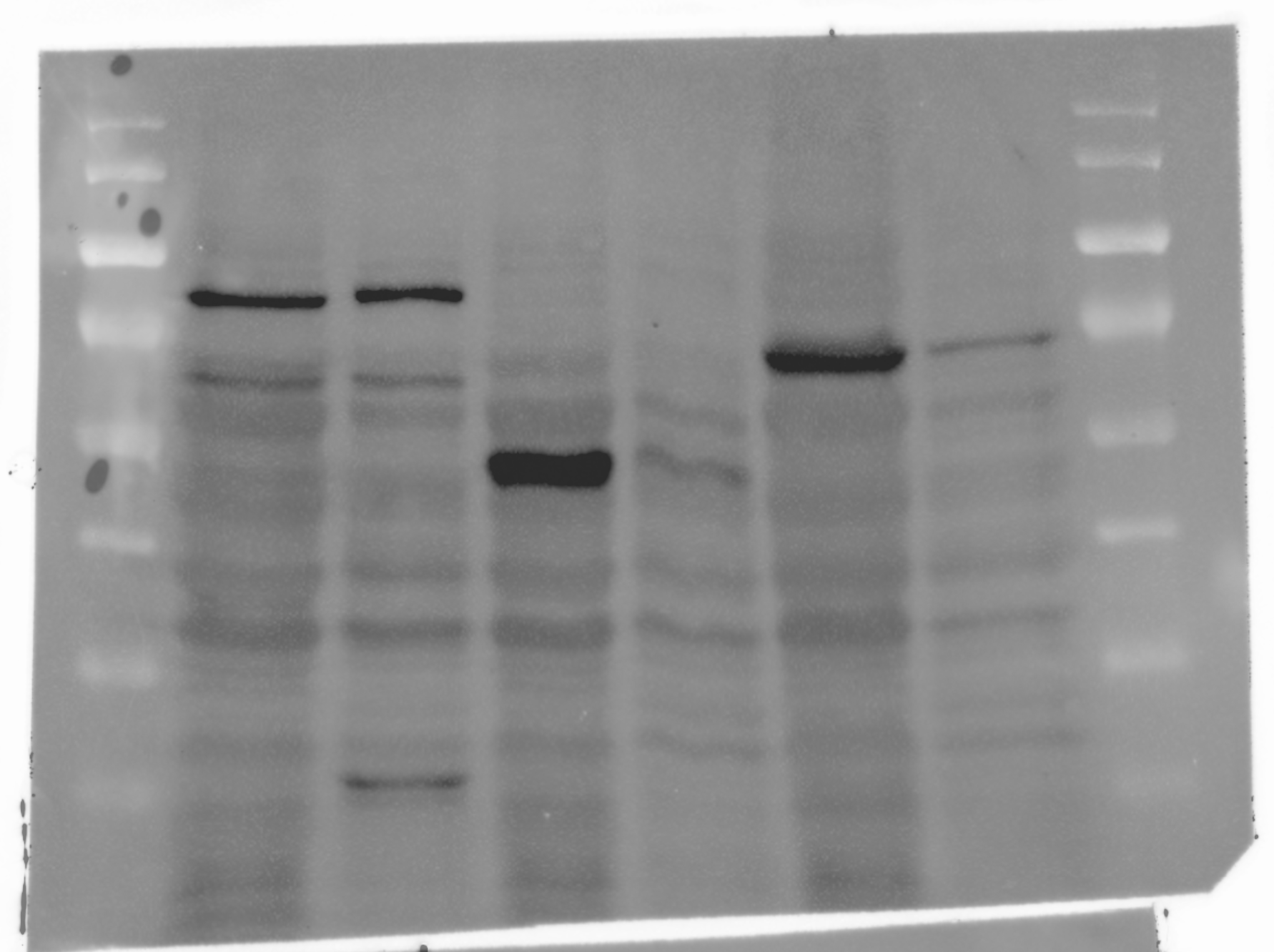

Supplement: Figure 3—figure supplement 1—source data 13. [file elife-89958-fig3-figsupp1-data13.zip › Figure3 Supplemental 1I Mam1GFP Western blot.tif]

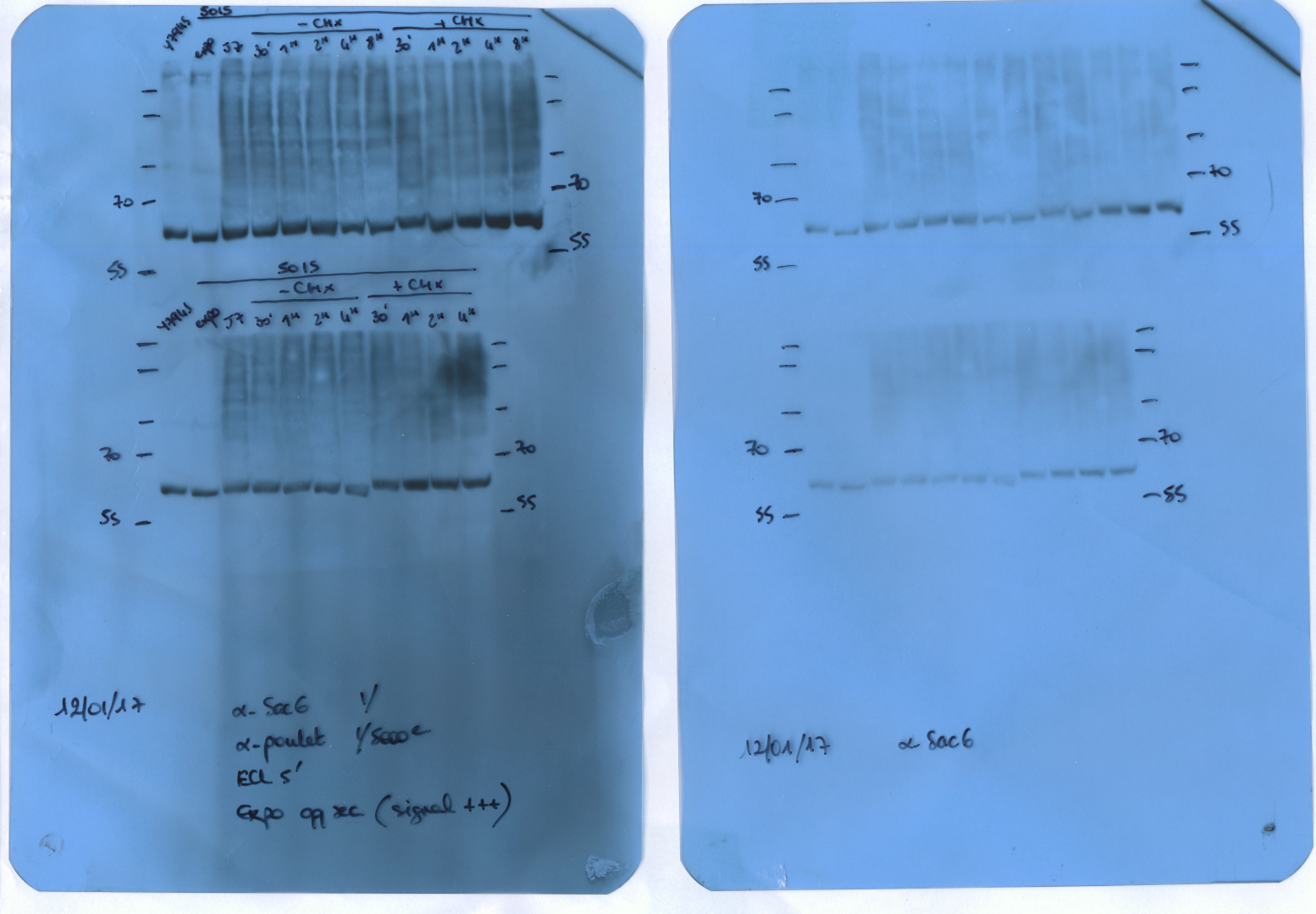

Supplement: Figure 5—figure supplement 1—source data 3. [file elife-89958-fig5-figsupp1-data3.zip › Figure 5 Supplemental 1C Western blot Sac6 control loading.tif]

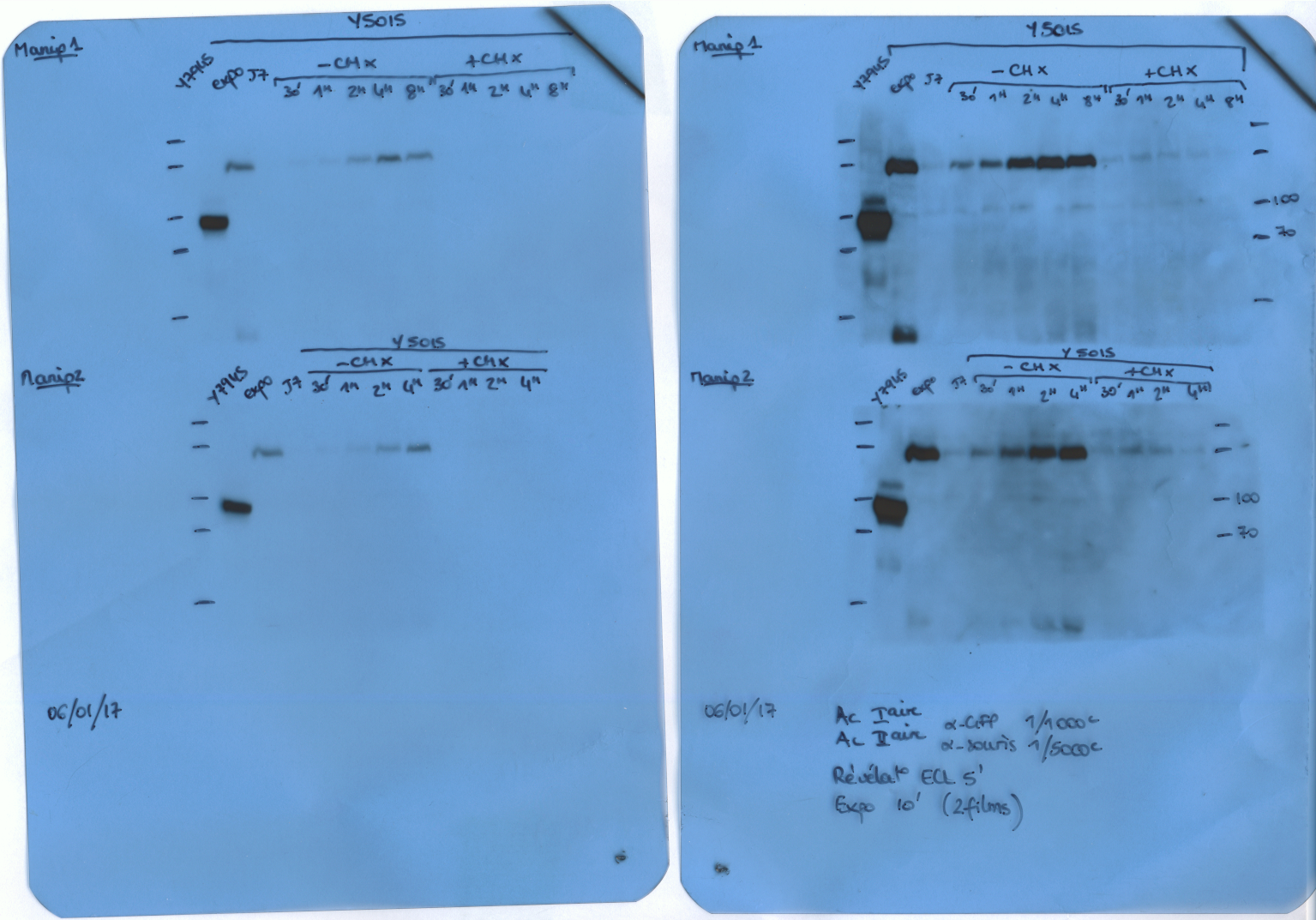

Supplement: Figure 5—figure supplement 1—source data 4. [file elife-89958-fig5-figsupp1-data4.zip › Figure 5 Supplemental 1C Western blot.tif]
