# Supplementary material for: A stable microtubule bundle formed through an orchestrated multistep process controls quiescence exit
Source: eLife. 2024 Mar 25;12:RP89958. doi: 10.7554/eLife.89958 (PMC10963028; doi:10.7554/eLife.89958)
Supplement: MDAR checklist [file elife-89958-mdarchecklist1.docx]

**Materials Design Analysis Reporting (MDAR)**

**Checklist for Authors**

The [MDAR framework](https://osf.io/xfpn4/) establishes a minimum set of requirements in transparent reporting mainly applicable to studies in the life sciences.

*eLife* asks authors to **provide detailed information within their article** to facilitate the interpretation and replication of their work. Authors can also upload supporting materials to comply with relevant reporting guidelines for health-related research (see [EQUATOR Network](http://www.equator-network.org/%20)), life science research (see the [BioSharing Information Resource](http://biosharing.org/)), or animal research (see the [ARRIVE Guidelines](http://www.plosbiology.org/article/info:doi/10.1371/journal.pbio.1000412) and the [STRANGE Framework](https://doi.org/10.1038/d41586-020-01751-5); for details, see *eLife*’s [Journal Policies](https://reviewer.elifesciences.org/author-guide/journal-policies)). Where applicable, authors should refer to any relevant reporting standards materials in this form.

For all that apply, please note **where in the article** the information is provided. Please note that we also collect information about data availability and ethics in the submission form.

**Materials:**

|  |  |  |
| --- | --- | --- |
| **Antibodies** | **Indicate where provided: section/figure legend** | **N/A** |
| a/ anti-GFP antibody (Abcam, Ab6556, RRID:AB_305564)  b/ anti-TagRFP antibody (Evrogen, Anti-tRFP antibody, AB233, gift from M. Rojo; RRID:AB_2571743)  c/ anti-Tat1 antibody (Merck, Hybridoma 00020911-DNA-5UG, gift from J-P. Javerzat)  d/ anti-alpha-tubulin (Abcam; YOL1/34 from rat, ref. Ab6161; RRID:AB_305329)  e/ anti-GFP antibody (Roche, anti-GFP mouse; ref. 11814460001; RRID:AB_390913)  f/ anti-myc antibody (Invitrogen, C-myc-tag (9E10); ref 13-2500;  RRID:AB_2533008)  g/ anti-Ade13 antibody (Homemade)  h/ anti-Act1 antibody (Homemade- gift from B. Goode)  i/ anti-Sac6 antibody (Homemade- gift from B. Goode) | a/ Figure1-figure supplement 1H  b/ Figure1-figure supplement 1H  c/ Figure2-figure supplement 1D,E  d/ Figure2-figure supplement 1A,B ; Figure3-figure supplement 1A  e/ Figure3-figure supplement 1I ; Figure5-figure supplement 1C  f/ Figure3-figure supplement 1E  g/ Figure1-figure supplement 1H ; Figure3-figure supplement 1E,I  h/ Figure3-figure supplement 1I  i/ Figure5-figure supplement 1C |  |
|  |  |  |
| **DNA and RNA sequences** | **Indicate where provided: section/figure legend** | **N/A** |
| Dad2UpXhoI - Forward - o4912 gtcCTCGAGATGGATTCAATAGATGAACAAATTGCTA  Dad2DoBamHI - Reverse o4913 gtcGGATCCacagcaccagcaccagcaccagcaccagcaccTTCGTTACCATCTACCCTAATTCTG  TUB3upxho - Forward - o3078 gccCTCGAGCCGTCGTGGAACCTTACAACACG  TUB3BamHIdown - Reverse - o3077  cGGggATCCccagcaccagcaccagcaccGAACTCCTCAGCGTAAGAATCG  Mam1F3xKpnI -Forward - o6010  acggtaccACAAGAAGTAATTTGAAAAAGTTACAAGA  Mam1R3xBamH1- Reverse - o6015  gtgggatccgccgcaccagcaccagcaccATTTTCATCTATATGTAGCTTTCTTTCC  Kar3F3x-1 - Forward - o5782 cacctcgagGAACTGGGTATGAAGGAGTTG  Kar3R3x - Reverse - o5773  ggtgggatccgccgcaccagcaccagcaccTTTTCTACTAACCAATCTGGTAGAATTCA  Tub3SacIup - Forward - o5679  CACGAGCTCGGTTCGATGGTTCATTAAACGTGG  Tub3BamHIrev- Reverse - o5680 GGCGGATCCGCCGCACCAGCACCAGCACCGAACTCCTCAGCGTAAGAATCG  ADH2pnot1 - Forward - o21-18 actGCGGCCGCtctctccggttacagcctgt  ADH2pRBcu1/spe1 - Reverse - o21-19 cagACTAGTtgtgtattacgatatagttaatagttg  FBcu1/Spe1mRuby2- Forward -o21-20actACTAGTatggtgtctaagggcgaagagc  TUB1+3UTRSal1- Reverse - o21-21actGTCGACccggcccagtttttgaacacatag | For integrative plasmid Dad2-3GFP construction (p5689 - pRS306 backbone) - Mat &Method section  For integrative plasmid Tub3-3GFP construction (p4553 - pRS306 backbone)- Mat &Method section  For integrative plasmid Mam1-3GFP construction (p6149 - pRS306 backbone)- Mat &Method section  For integrative plasmid Kar3-3GFP construction (p6032 - pRS305 backbone)- - Mat &Method section  For integrative plasmid Tub3-tdimer construction (p5991 - pRS306 backbone)- Mat &Method section  For integrative plasmid pRS303-*ADH2p-mRuby2-Tub1* construction (pMC152 - pRS303 backbone)- Mat &Method section |  |
|  |  |  |
| **Yeast strains** | **Indicate where provided: section/figure legend** | **N/A** |
| - Y11747- Spc42-mRFP1-KANR; mTurquoise2-Tub1-Leu2;his3Δ1; leu2Δ0; met15Δ0; ura3Δ0 Mata (this study)  - Isy53 - mTurquoise2-Tub1-Leu2, Nuf2-GFP-His, his3Δ1; leu2Δ0; ura3Δ0;Matα(this study)  - Y11741 - Spc42-mRFP1-KANR; mTurquoise2-Tub1-Leu2;Stu2-GFP-HIS3;his3Δ1; leu2Δ0; met15Δ0; ura3Δ0, Mata(this study)  - Y12182- Tub4-mTurquoise2-URA3;his3Δ1; leu2Δ0; met15Δ0; ura3Δ0, Mata (this study)  - Y11911 - W303 - bar1, ade2-1? trp1-1? lys2? leu2-3,112, his3-11,15, ura3-1  tub4::Tub4-mTurquoise2-URA3MX (from S. Jaspersen, Elife, doi:10.7554/eLife.08586.)  - Isy335- his3Δ1; leu2Δ0; met15Δ0; ura3Δ0, mTurquoise2-Tub1::LEU2; ADH2p-mruby2-Tub1::HIS3, Mata (this study)  - Isy849 his3Δ1; leu2Δ0; met15Δ0; ura3Δ0, ADH2p-mruby2-Tub1::HIS3, Mata(this study)  - Y10626 his3Δ1; leu2Δ0; met15Δ0; ura3Δ0, mTurquoise2-Tub1-Leu2, MAta(this study)  - Y12195 mTurquoise2-Tub1-Leu2; TUB3-3xGFP-URA3;his3Δ1; leu2Δ0; met15Δ0; ura3Δ0, Mata (this study)  - Y12230 - mTurquoise2-Tub1-Leu2;his3Δ1; leu2Δ0; lys2Δ0; ura3Δ0, Matα (This study)  - Y10626 his3Δ1; leu2Δ0; met15Δ0; ura3Δ0 mTurquoise2-Tub1-Leu2 Mata (This study)  - Y12244 - mTurquoise2-Tub1-Leu2/TUB1;his3Δ1/his3Δ1; leu2Δ0/leu2Δ0; lys2Δ0/LYS2; met15Δ0/MET15; ura3Δ0/ura3Δ0, Mata/α (this study)  - Y12245 - mTurquoise2-Tub1-Leu2/mTurquoise2-Tub1-Leu2;  his3Δ1/his3Δ1; leu2Δ0/leu2Δ0; lys2Δ0/LYS2; met15Δ0/MET; ura3Δ0/ura3Δ0 Mata/α (this study)  - Isy125 Tub4-mTurquoise2-URA3; mRUBY2-Tub1-LEU2;his3Δ1; leu2Δ0; met15Δ0; ura3Δ0 Mata (this study)  - Y11997 - HIS3p:mRUBY2-Tub1-LEU2; NUF2-GFP-HIS3;his3Δ1; leu2Δ0; met15Δ0; ura3Δ0, Mata (this study)  - Y12276 - HIS3p:mRUBY2-Tub1-LEU2; NUF2-GFP-HIS3; NUP2-tdimer-URA3;  his3Δ1; leu2Δ0; met15Δ0; ura3Δ0, Mata (this study)  - Y10907 - Dad2-GFP-HIS3; mRUBY2-Tub1-LEU2; his3Δ1; leu2Δ0; met15Δ0; ura3Δ0, Mata (this study)  - Y12195 - mTurquoise2-Tub1-Leu2; TUB3-3xGFP-URA3;his3Δ1; leu2Δ0; met15Δ0; ura3Δ0, Mata (this study)  - Y12264 - yomWasabi-Tub1-Leu2; TUB3-tdimer-URA3;his3Δ1; leu2Δ0; met15Δ0; ura3Δ0, Mata (this study)  - Isy693 - mTurquoise2-Tub1-LEU2 ; ura3-52, his3D200, leu2D1, trp1D63, met-, Matα (this study)  - Isy694- mTurquoise2-Tub1-LEU2 ; ura3-52, his3D200, leu2D1, trp1D63, met- Matα (this study)  - Y12051 - mTurquoise2-Tub1-Leu2;ura3-52, his3D200, leu2D1, trp1D63, met Mata (this study)  - Y12052 - tub3Δ::URA3; mTurquoise2-Tub1-Leu2; his2D200; leu2D1; trp1D63, met (tub3delta )Mata (this study)  - Y12137 - Nuf2-GFP-HIS3; mTurquoise2-Tub1-Leu2;tub3Δ::URA3; his2 Δ200; leu2D1; trp1D63 (tub3delta)Matα (this study)  - Y12138 Nuf2-GFP-HIS3; mTurquoise2-Tub1-Leu2;tub3Δ::URA3; his2D200; leu2D1; trp1D63 (tub3delta)Matα (this study)  - Isy702 - mTurquoise2-Tub1-LEU2 ; ura3-52, his3D200, leu2D1, trp1D63, met-, tub3::TUB1 (tub1only) Matα (this study)  - Isy57 - ndc80-1, mTurquoise2-Tub1-Leu2, Nuf2-GFP-His, his3Δ1; leu2Δ0; lys2Δ0; ura3Δ0; Mat? (this study)  - Isy866 - ipl1::KanMX6 :: ipl1-as5-MYC :: HIS3 :: LEU2, mTurquoise2-Tub1-Leu2,  his3Δ1/his3-11,15? ; leu2Δ0/leu2-3; lys2Δ0; ura3Δ0/ura3-1? , trp1-1?, can1-100? Mat? (this study)  - Isy83 - sli15-GFP-HIS3; mTurquoise2-Tub1-Leu2;leu2Δ0; ura3Δ0; his3Δ1, Matα (this study)  - Isy81 - Bir1-GFP-HIS3; mTurquoise2-Tub1-Leu2;his3Δ1; leu2Δ0; met15Δ0; ura3Δ0, Mata (this study)  - Isy12- NUF2 -GFP-HIS3; bim1::KanR; mTurquoise2-Tub1-Leu2;his3Δ1; leu2Δ0; met15Δ0; ura3Δ0, Mata (this study)  - Isy113- bir1::kanMX4; mTurquoise2-Tub1-Leu2;his3Δ1; leu2Δ0; MET15/met15Δ0; lys2Δ0/LYS2; ura3Δ0, Mata (this study)  - Isy189 - bir1::kanMX4; mTurquoise2-Tub1-Leu2; bim1::HIS3;his3Δ1; leu2Δ0; MET15/met15Δ0; lys2Δ0/LYS2; ura3Δ0, Mata (this study)  - Isy345 - Slk19-GFP-HIS3; mTurquoise2-Tub1-Leu2, his3Δ1; leu2Δ0; met15Δ0; ura3Δ0;  Mata (this study)  - Isy115 - slk19::KanR; mTurquoise2-Tub1-Leu2;his3Δ1; leu2Δ0; lys3D0; ura3Δ0, Matα (this study)  - Isy126 - bim1::KanR; slk19::KanR; NUF2 -GFP-HIS3; his3Δ1; leu2Δ0; ura3Δ0, Matα (this study)  - Isy179 - mam1::KanR; mTurquoise2-Tub1-Leu2; his3Δ1; leu2Δ0; lys2Δ0; ura3Δ0 Matα (this study)  - Isy170 - csm1::KanR; mTurquoise2-Tub1-Leu2; his3Δ1; leu2Δ0; lys2Δ0; ura3Δ0 Matα (this study)  - Isy178 - spo13::KanR; mTurquoise2-Tub1-Leu2; his3Δ1; leu2Δ0; lys2Δ0; ura3Δ0 Matα (this study)  - Isy180 - lrs4::KanR; mTurquoise2-Tub1-Leu2; his3Δ1; leu2Δ0; lys2Δ0; ura3Δ0 Matα (this study)  - Isy268 - lrs4::KanR;bim1::KanR; mTurquoise2-Tub1-Leu2; NUF2 -GFP-HIS3; his3Δ1; leu2Δ0; met15Δ0?; lys2Δ0? Mata (this study)  - Isy270 - mam1::KanR; bim1::KanR; mTurquoise2-Tub1-Leu2;NUF2 -GFP-HIS3;  his3Δ1; leu2Δ0; lys2Δ0 ?; met15Δ0?; ura3Δ0, Mat? (this study)  - Isy272 - csm1::KanR; bim1::KanR; mTurquoise2-Tub1-Leu2; NUF2 -GFP-HIS3; his3Δ1; leu2Δ0; met15Δ0?;lys2Δ0?; ura3Δ0; Matα (this study)  - Isy276 - spo13::KanR; bim1::KanR; mTurquoise2-Tub1-Leu2; NUF2 -GFP-HIS3; his3Δ1; leu2Δ0; met15Δ0?; lys2Δ0? Mata (this study)  - Isy353 - spo13::KanR; bim1::KanR; mTurquoise2-Tub1-Leu2; NUF2 -GFP-HIS3; Nup2-tdimer-URA3; his3Δ1; leu2Δ0; met15Δ0?; lys2Δ0? Mata (this study)  - Isy358 - spo13::KanR; mTurquoise2-Tub1-Leu2; Nup2-tdimer-URA3, his3Δ1; leu2Δ0; lys2Δ0; ura3Δ0 Matα (this study)  - Isy352 - mam1::KanR; mTurquoise2-Tub1-Leu2; Nup2-tdimer-URA3; his3Δ1; leu2Δ0; lys2Δ0; ura3Δ0 Matα (this study)  - Isy356 - mam1::KanR; bim1::KanR;mTurquoise2-Tub1-Leu2;NUF2 -GFP-HIS3;Nup2-tdimer-URA3 his3Δ1; leu2Δ0; lys2Δ0 ?; met15Δ0?; ura3Δ0 Mat? (this study)  - Isy355 - csm1::KanR; bim1::KanR;mTurquoise2-Tub1-Leu2; NUF2 -GFP-HIS3;Nup2-tdimer-URA3;his3Δ1; leu2Δ0; met15Δ0?;lys2Δ0?; ura3Δ0; Matα (this study)  - Isy357 - csm1::KanR; mTurquoise2-Tub1-Leu2; Nup2-tdimer-URA3 his3Δ1; leu2Δ0; lys2Δ0 ?; met15Δ0?; ura3Δ0 Mata (this study)  - Isy351 - lrs4::KanR; bim1::KanR; mTurquoise2-Tub1-Leu2; NUF2 -GFP-HIS3;Nup2-tdimer-URA3; his3Δ1; leu2Δ0; met15Δ0?; lys2Δ0? Mata (this study)  - Isy359 - lrs4::KanR; mTurquoise2-Tub1-Leu2; Nup2-tdimer-URA3; his3Δ1; leu2Δ0; lys2Δ0; ura3Δ0 Matα (this study)  - Y10907 - Dad2-GFP-HIS3; mRUBY2-Tub1-LEU2; his3Δ1; leu2Δ0; met15Δ0; ura3Δ0 Mata (this study)  - Isy248 - his3Δ1; leu2Δ0; met15Δ0; ura3Δ0 ipl1-1, mTurquoise2-Tub1-Leu2; Mata (this study)  - Isy844 - mTurquoise2-Tub1-Leu2, Nuf2-GFP-His, his3Δ1; leu2Δ0; ura3Δ0;  sli15(L656S):Hygromycin (sli15-3) Matα (this study)  - Y11993 - SPC42-mRFP1-KANR; mTurquoise2-Tub1-Leu2; BIM1-3xGFP-URA3;  his3Δ1; leu2Δ0; met15Δ0; ura3Δ0, Mata (this study)  - Isy151 KAR3-3GFP-LEU2; mTurquoise2-Tub1-HIS3;his3Δ1; leu2Δ0; met15Δ0; ura3Δ0 Mata (this study)  - Isy218 - kar3::KanR; his3Δ1; leu2Δ0; met15Δ0; ura3Δ0; mTurquoise2-Tub1-Leu2, Mata (this study)  - Y11867 - cik1::KanR; mTurquoise2-Tub1-LEU2;his3Δ1; leu2Δ0; met15Δ0; ura3Δ0, Matα (this study)  - Y11720 - vik1::kanR; mTurquoise2-Tub1-LEU2;leu2Δ0, his3Δ1, lys2Δ0, ura3Δ0 Matα (this study)  - Isy142 - kip1::kanMX4; Nuf2-GFP-HIS3; mTurquoise2-Tub1-Leu2;leu2Δ0, his3Δ1, lys2Δ0, ura3Δ0 Matα (this study)  - Isy143 - Cin8::KanMX4; Nuf2-GFP-HIS3; mTurquoise2-Tub1-Leu2; his3Δ1; leu2Δ0; lys2Δ0; ura3Δ0 Matα (this study)  - Y12234 - kip3::KanR; mTurquoise2-Tub1-Leu2;his3Δ1; leu2Δ0; met15Δ0; ura3Δ0 Mata (this study)  - Isy241 - Kip2::KanR; Nuf2-GFP-HIS3;his3Δ1; leu2Δ0; met15Δ0; ura3Δ0, mTurquoise2-Tub1-Leu2; Mata (this study)  - Y10725 - his3Δ1; leu2Δ0; met15Δ0; ura3Δ0 ; mEOS2-Tub1-Leu2 ; BIM1 3XEGFP-Ura3, Mata (this study)  - Isy172 - cin8::KanMX4; Nuf2-GFP-HIS3; mEOS2-Tub1-Leu2;his3Δ1; leu2Δ0; lys2Δ0; ura3Δ0, Matα (this study)  - Isy82 - KIP1-GFP-HIS3; mTurquoise2-Tub1-Leu2;his3Δ1; leu2Δ0; met15Δ0; ura3Δ0, Mata (this study)  - Isy347 - Cin8-GFP-HIS3; mTurquoise2-Tub1-Leu2, his3Δ1; leu2Δ0; met15Δ0; ura3Δ0; Mata (this study)  - Isy707 - bim1::KanR ; kip1::kanMX4; Nuf2-GFP-HIS3; mTurquoise2-Tub1-Leu2;  leu2Δ0, his3Δ1, lys2Δ0, ura3Δ0, Mata (this study)  - Y11722 - SPC42-mRFP1-KanR; Nuf2-GFP-HIS3;his3Δ1; leu2Δ0; lys2Δ0; ura3Δ0, Matα (this study)  - Y5247 - his3Δ1; leu2Δ0; met15Δ0; ura3Δ0; STU2-GFP-HIS3 (ThermoFisher Scientific (Waltham, MA, USA)  - Isy181 kip3::KanR; she1::HIS; mTurquoise2-Tub1-Leu2; Nuf2-GFP-HIS3; leu2Δ0, ura3Δ0; his3Δ1; met15Δ0 Matα (this study)  - Isy152 - she1::HIS; mTurquoise2-Tub1-Leu2;his3Δ1; leu2Δ0; met15Δ0; ura3Δ0 Mata (this study)  - Isy155 - cdh1::HIS; mTurquoise2-Tub1-Leu2;ura3-52, his3D200, leu2D1, trp1D63, met-  Matα (this study)  - Isy157 - dcc1::HIS; mTurquoise2-Tub1-Leu2;ura3-52, his3D200, leu2D1, trp1D63, met-, Matα (this study)  - Isy198 - tor1::KanR; his3Δ1; leu2Δ0; ura3Δ0; met15Δ0 ; mTurquoise-Tub1-UTR::Leu2, Mata (this study)  - Y11025 - kip3::KanR; Nuf2-GFP-HIS3; met15Δ0, leu2Δ0, ura3Δ0; his3Δ1, Matα (this study)  - Isy289 kip3::KanR; she1::HIS; mTurquoise2-Tub1-Leu2; Nuf2-GFP-HIS3; Spc42-tdimer  leu2Δ0, ura3Δ0; his3Δ1; met15Δ0, Matα (this study)  - Isy760 - Spc42-tdimer-URA3; his3Δ1; leu2Δ0; lys2Δ0; ura3Δ0, mTQ2-TUB1::LEU, Matα (this study)  - Y12277 - mTurquoise2-Tub1-Leu2, ndc10-1, his3Δ1; leu2Δ0; met15Δ0; ura3Δ0; Mata (this study)  - Isy254 - ipl1-2; mTurquoise2-Tub1-Leu2; his3Δ1; leu2Δ0; met15Δ0??? ura3Δ0 ade16 ade17(this study)  - Isy10 - ndc80-1; mTurquoise2-TUB1-LEU2;his3Δ1; leu2Δ0; met15Δ0; ura3Δ0 Mata (this  study)  - Y11766 - cdc14-1; mTurquoise2-Tub1-Leu2; Spc42-mRFP-KanR;his3Δ1; leu2Δ0; ura3Δ0 Mata (this study)  - Isy188 - cdc15-1; mTurquoise2-Tub1-Leu2; his3Δ1; leu2Δ0; met15Δ0; ura3Δ0 Mata (this study) | Figure 1A, 3D, 3H, 4B, Figure 5 Supplemental1E  Figure 1A,B,C,H,2A,B, 3F, 4B, 4C, 4H;5C Figure 1-Supplemental A,D,E; Figure 3 Supplemental1F; Figure 5 Supplemental1G  Figure 1D, Figure 1-Supplemental E  Figure 1E  Figure 1-Supplemental1F  Figure 1F  Figure 1F  Figure 1F, Figure 1-Supplemental J  Figure 5-Supplemental 1E  Figure 1-Supplemental B  Figure 1-Supplemental C  Figure 1-Supplemental C  Figure 1-Supplemental C  Figure 1-Supplemental G  Figure 3I ; Figure 1-Supplemental I  Figure 3-SupplementalA  Figure 1-Supplemental K  Figure 1-Supplemental K  Figure 2A, Figure 2-Supplemental A  Figure 2A  Figure 2B  Figure 2B  Figure 2B  Figure 2B  Figure 2B  Figure 2B  Figure 2B  Figure 3A  Figure 3B    Figure 3C  Figure 3C  Figure 3D  Figure 3D  Figure 3D  Figure 3E  Figure 3F  Figure 3F  Figure 3H  Figure 3H  Figure 3H  Figure 3H  Figure 3H  Figure 3H  Figure 3H  Figure 3H  Figure 3H  Figure 3H  Figure 3H  Figure 3G  Figure 3G  Figure 3G  Figure 3G  Figure 3G  Figure 3 Supplemental1A  Figure 3 Supplemental1C  Figure 3 Supplemental1F  Figure 3 Supplemental1G  Figure 4A  Figure 4B  Figure 4B  Figure 4B  Figure 4H  Figure 4C,D  Figure 4H  Figure 4 Supplemental1F  Figure 4E  Figure 4E  Figure 4F  Figure 4 Supplemental1B  Figure 4 Supplemental1E  Figure 5A  Figure 5A  Figure 5C  Figure 5C  Figure 5 Supplemental1D  Figure 5 Supplemental1D  Figure 5 Supplemental1D  Figure 5D  Figure 5E,F  Figure 5E,F  Figure 5 Supplemental1F  Figure 5 Supplemental1G  Figure 5 Supplemental1G  Figure 5 Supplemental1E  Figure 5 Supplemental1E |  |

**Design:**

| **Study protocol** | **Indicate where provided: section/figure legend** | **N/A** |
| --- | --- | --- |
| If the study protocol has been pre-registered, provide DOI. For clinical trials, provide the trial registration number OR cite DOI. |  | N/A |
|  |  |  |
| **Laboratory protocol** | **Indicate where provided: section/figure legend** | **N/A** |
| Provide DOI OR other citation details if detailed step-by-step protocols are available. |  | N/A |
|  |  |  |
| **Experimental study design (statistics details) *** | | |
| **For in vivo studies: State whether and how the following have been done** | **Indicate where provided: section/figure legend. If it could have been done, but was not, write “not done”** | **N/A** |
| Sample size determination |  | N/A |
| Randomisation |  | N/A |
| Blinding |  | N/A |
| Inclusion/exclusion criteria |  | N/A |
|  |  |  |
| **Sample definition and in-laboratory replication** | **Indicate where provided: section/figure legend** | **N/A** |
| State number of times the experiment was replicated in the laboratory. | The number of experiments and their size are indicated in the legend to each figure. |  |
| Define whether data describe technical or biological replicates. | All the data are biological replicates, as they come from differents cultures |  |
|  |  |  |

**Analysis:**

| **Attrition** | **Indicate where provided: section/figure legend** | **N/A** |
| --- | --- | --- |
| Describe whether exclusion criteria were pre-established. Report if sample or data points were omitted from analysis. If yes, report if this was due to attrition or intentional exclusion and provide justification. |  | N/A |
|  |  |  |
| **Statistics** | **Indicate where provided: section/figure legend** | **N/A** |
| Describe statistical tests used and justify choice of tests. | Student's T-test was used to compare two different populations. Anova (Prism Sowtware) was used if more than two value distributions were to be compared. |  |
|  |  |  |
| **Data availability** | **Indicate where provided: section/submission form** | **N/A** |
| For newly created and reused datasets, the manuscript includes a data availability statement that provides details for access (or notes restrictions on access). |  | N/A |
| When newly created datasets are publicly available, provide accession number in repository OR DOI and licensing details where available. |  | N/A |
|  |  |  |
| **Code availability** | **Indicate where provided: section/figure legend** | **N/A** |
| For any computer code/software/mathematical algorithms essential for replicating the main findings of the study, whether newly generated or re-used, the manuscript includes a data availability statement that provides details for access or notes restrictions. |  | **N/A** |
| Where newly generated code is publicly available, provide accession number in repository, OR DOI OR URL and licensing details where available. State any restrictions on code availability or accessibility. |  | **N/A** |
| If reused code is publicly available provide accession number in repository OR DOI OR URL, OR citation. |  | **N/A** |

**Reporting:**

The MDAR framework recommends adoption of discipline-specific guidelines, established and endorsed through community initiatives.

| **Adherence to community standards** | **Indicate where provided: section/figure legend** | **N/A** |
| --- | --- | --- |
| State if relevant guidelines (e.g., ICMJE, MIBBI, ARRIVE, STRANGE) have been followed, and whether a checklist (e.g., CONSORT, PRISMA, ARRIVE) is provided with the manuscript. |  | **N/A** |
